# Suspended scattering particles in motion using OCT angiography in branch retinal vein occlusion disease cases with cystoid macular edema

**DOI:** 10.1038/s41598-020-70784-7

**Published:** 2020-08-19

**Authors:** Kwang-Eon Choi, Sangheon Han, Cheolmin Yun, Seong-Woo Kim, Jaeryung Oh

**Affiliations:** 1 Department of Ophthalmology, Korea University College of Medicine, Seoul, South Korea; 2 Department of Chemistry, University of Illinois at Urbana-Champaign, Champaign, IL USA

**Keywords:** Retinal diseases, Blood-brain barrier, Molecular medicine

## Abstract

We aimed to investigate the clinical implication of suspended scattering particles in motion (SSPiM) using optical coherence tomography angiography (OCTA) among branch retinal vein occlusion disease (BRVO) cases with macular edema (ME). Medical records of BRVO patients were reviewed. Central retinal thickness (CRT), ME type, and cyst size on optical coherence tomography images were evaluated before and after intravitreal bevacizumab injection. Nonperfusion area, SSPiM, and microvascular abnormalities in OCTA images were evaluated using a Heidelberg machine. SSPiM was identified in 24 of 56 cases. There were no differences in baseline characteristics between groups with and without SSPiM. Disease duration, disease-free duration, previous injection number, microaneurysms in the superficial vascular complex, and microaneurysms in the deep vascular complex (DVC) (*p* = 0.003, 0.013, 0.028, 0.003, < 0.001, respectively) differed significantly between the two groups. After multivariate logistic analysis, microaneurysms in the DVC were the only different factor between the two groups (odds ratio [OR]: 0.091; *p* = 0.001). Furthermore, SSPiM in the DVC (OR 10.908; *p* = 0.002) and nonperfusion grade (OR 0.039; *p* < 0.001) were significantly associated with cyst response after intravitreal injection. SSPiM may be correlated with microaneurysms in the DVC and a poor anatomical response after intravitreal injection.

## Introduction

Branch retinal vein occlusion (BRVO) is a major vasculopathy among retinal diseases, and when it occurs, macular edema (ME) is also usually present and well-developed^1,2^. Optical coherence tomography (OCT) and fluorescein angiography are widely used to assess various ocular diseases^3–5^. However, OCT angiography (OCTA), which is a more recent technology, has greatly helped to elucidate differences in structural anomalies and identify anatomical vascular patterns not previously seen before^6–12^.

OCTA images are acquired by analyzing differences in reflectivity from repeated OCT B-scan images^13,14^. Microvascular abnormalities such as microaneurysms, telangiectasia, and neovascularization can be assessed in OCTA images in addition to areas of nonperfusion as well as areas of vascular density. In most cases, distinction between areas of nonperfusion and vascular density is possible, but nonvascular decorrelation signals or artifacts may prevent such a distinction^15–17^. In one study of nonvascular decorrelation signals, Kashani et al. proposed the concept of suspended scattering particles in motion (SSPiM)^18^. They suggested that severe breakdown of the blood-retinal barrier (BRB) induces an increase in particle efflux and that SSPiM appear due to a decorrelation signal caused by Brownian motion of particles in intraretinal cysts. They also demonstrated experimentally that an intralipid solution generated SSPiM-like signals^18^. In a previous study, we focused on and evaluated decorrelation signals like SSPiM in diabetic ME (DME) patients using OCTA images, and found that both steroid and anti-vascular endothelial growth factor (VEGF) treatment responses were poor in eyes that had SSPiM^19^. Although both BRVO and diabetic retinopathy are characterized by edematous maculopathy, there are differences in the pathophysiology of these two vascular diseases.

In the present study, we compared the basic characteristics of BRVO cases with and without SSPiM. We also examined treatment responses in these two groups of patients.

## Results

### Patient characteristics

Among the total 135 patients identified during the chart review period, 43 cases were excluded due to no history of intravitreal injections after symptomatic macular edema, and another 36 cases were excluded due to inadequate image quality and follow-up loss. Finally, 56 cystoid ME cases treated with intravitreal Avastin injection were included. Thirty-two cases did not show SSPiM, and 24 cases showed SSPiM. Mean age was 62.35 ± 9.92 years, and 34 patients were females. Mean disease duration was 10.74 ± 15.24 months, and mean disease-free duration was 6.43 ± 10.24 months. All cases received at least one injection, and the mean previous injection number was 2.43 ± 2.53. Mean follow-up duration was 8.75 ± 3.48 months.

### OCT and OCTA findings

Patients with SSPiM (group 1) showed no significant differences in initial vision, age, sex, intraocular pressure (IOP), ocular perfusion pressure (OPP), or central retinal thickness (CRT) compared with patients without SSPIM (group 2). Group 1 had a longer disease duration (16.13 ± 17.52 months vs 6.69 ± 12.04 months, *p* = 0.003 by Mann–Whitney U test), longer disease-free duration (7.29 ± 8.93 months vs 5.78 ± 11.22 months, *p* = 0.013 by Mann–Whitney U test), and a greater number of previous injections (3.08 ± 2.26 vs 1.91 ± 2.64, *p* = 0.028 by Mann–Whitney U test) than group 2. Group 1 showed a greater tendency for microaneurysms in the superficial vascular complex (SVC, yes/no; 19/5 vs 12/20; *p* = 0.002) and microaneurysms in the deep vascular complex (DVC, yes/no; 20/4 vs 10/22; *p* = 0.001) than group 2. All patients had telangiectasia in the SVC and most patients had telangiectasia in the DVC. None of the cases had definite collateral vessels in either the SVC or DVC. After one intravitreal bevacizumab injection, mean CRT showed no significant difference between the two groups. Cystoid changes after treatment in group 1 were less extensive than those in group 2 (Table 1).

**Table 1:** Comparison of basic characteristics between group 1 (patients with suspended scattering particles in motion (SSPiM)) and group 2 (those without SSPiM).

	All (n = 56)	Group 1 (with SSPiM) (n = 24)	Group 2 (without SSPiM) (n = 32)	*p*
Age (years)	62.35 ± 9.92	61.79 ± 9.07	62.78 ± 10.65	0.703^a^
Sex (M/F)	22/34	9/15	13/19	1.000^b^
Total disease duration (months)	10.74 ± 15.24	16.13 ± 17.52	6.69 ± 12.04	**0**.**003**^a^
Disease-free duration (months)	6.43 ± 10.24	7.29 ± 8.93	5.78 ± 11.22	**0**.**013**^a^
Follow-up duration (months)	8.75 ± 3.48	8.13 ± 3.31	9.22 ± 3.57	0.063^a^
Previous injection number	2.43 ± 2.53	3.08 ± 2.26	1.91 ± 2.64	**0**.**028**^a^
Subsequent injection number	1.20 ± 0.92	1.38 ± 1.01	0.94 ± 0.76	0.113^a^
IOP (mmHg)	15.50 ± 2.68	15.83 ± 2.37	15.25 ± 2.90	0.429^**a**^
MOPP (mmHg)	55.69 ± 5.53	56.63 ± 6.39	54.99 ± 4.77	0.220^**a**^
Visual acuity-pre (logMAR)	0.53 ± 0.54	0.48 ± 0.63	0.57 ± 0.46	0.096^**a**^
Visual acuity-post (logMAR)	0.35 ± 0.39	0.27 ± 0.35	0.41 ± 0.40	0.055^**a**^
Visual acuity (improved/decreased)	34/22	14/10	20/12	0.788^**a**^
ME type (cystoid ME/cystoid ME + sRF)	34/22	18/6	16/16	0.096^b^
Nonperfusion grade (1/2)	32/24	13/11	19/13	0.788^b^
Telangiectasia in the SVC (Y/N)	56/0	24/0	32/0	
Telangiectasia in the DVC (Y/N)	54/2	22/2	32/0	0.179^c^
Microaneurysms in the SVC (Y/N)	31/25	19/5	12/20	**0**.**003**^b^
Microaneurysms in the DVC (Y/N)	30/26	20/4	10/22	** < 0**.**001**^d^
Pre-CRT (μm)	427.55 ± 128.10	392.17 ± 118.03	454.09 ± 130.69	0.076^**a**^
Post-CRT (μm)	321.14 ± 85.75	329.25 ± 79.22	315.07 ± 91.11	0.253^**a**^
Responder/Nonresponder (10% reduction in CRT) (Y/N)	40/16	18/6	22/10	0.767^b^
Intraretinal cyst size-pre (mm^2^)	0.16 ± 0.12	0.19 ± 0.12	0.14 ± 0.11	0.140^**a**^
Intraretinal cyst size-post (mm^2^)	0.06 ± 0.11	0.10 ± 0.13	0.03 ± 0.09	** < 0**.**001**^**a**^
Ratio of cyst change	0.72 ± 0.42	0.45 ± 0.45	0.90 ± 0.29	** < 0**.**001**^**a**^
Responder/nonresponder (20% reduction in cyst size) (Y/N)	46/10	16/8	30/2	**0**.**013**^**d**^

*SSPiM* suspended scattering particles in motion, *M* male, *F* female, *IOP* intraocular pressure, *MOPP* mean ocular perfusion pressure, *logMAR* log of the minimum angle of resolution, *ME* macular edema, *sRF* subretinal fluid, *SVC* superficial vascular complex, *DVC* deep vascular complex, *Y* yes, *N* no, *CRT* central retinal thickness.

^a^Mann–Whitney U test.

^b^Pearson K square test.

^c^Linear-by-linear test.

^d^Fisher’s exact test.

In univariate binary logistic regression analysis, longer total disease duration (odds ratio [OR]: 0.950; *p* = 0.045), presence of microaneurysms in the SVC (OR 0.158; *p* = 0.003), and presence of microaneurysms in the DVC (OR 0.091; *p* < 0.001) were significantly associated with the presence of SSPiM. In multivariate logistic regression analysis, only the presence of microaneurysms in the DVC (OR 0.091; *p* < 0.001) was significantly associated with the presence of SSPiM (Table 2).

**Table 2:** Binary logistic regression analysis of factors associated with the presence of suspended scattering particles in motion.

	Univariate binary logistic regression analysis			Multivariate binary logistic regression analysis		
	OR	β	*p* value	OR	β	*p* value
Total disease duration	0.950	− 0.051	0.045			0.071
Disease free duration	0.963	− 0.038	0.223			0.292
Previous injection number	0.809	− 0.211	0.109			0.071
Microaneurysm in SVC	0.158	− 1.846	0.003			0.078
Microaneurysm in DVC	0.091	− 2.398	< 0.001	0.091	− 2.398	** < 0**.**001**

*OR* odds ratio, *SVC* superficial vascular complex, *DVC* deep vascular complex.

Cyst non-responders to anti-VEGF treatment had a wider angle of the nonperfusion area in the fovea 3-mm zone than responders (grade 1/grade 2, 1/9 vs 31/15, *p* = 0.001). SSPiM in the SVC (yes/no, 6/4 vs 11/35, *p* = 0.034), and SSPiM in the DVC (yes/no, 8/2 vs 16/30, *p* = 0.013 by Fisher’s exact test) were more frequently detected in cyst nonresponders than responders. However, multivariate logistic regression analysis using nonperfusion grade, SSPiM in the SVC, and SSPiM in the DVC showed that SSPiM in the DVC (OR 10.908; *p* = 0.013) and severe nonperfusion grade (OR 0.039; *p* = 0.006) were significantly more likely to be detected in cyst nonresponders after a single intravitreal injection than cyst responders (Table 3). There were significant differences in visual acuity (post) (logMAR) (0.27 ± 0.27 vs 0.55 ± 0.54, *p* = 0.027), post-injection cyst size (0.03 ± 0.07 mm^2^ vs 0.12 ± 0.16 mm^2^, *p* = 0.006), and cyst size change (0.80 ± 0.37 ratio vs. 0.53 ± 0.48 ratio, *p* = 0.013) between CRT responders and nonresponders by Mann–Whitney U test. However, there were no significant differences in baseline factors between CRT responder and CRT nonresponders.

**Table 3:** Binary logistic regression analysis of cyst treatment response.

	Univariate binary logistic regression analysis			Multivariate binary logistic regression analysis		
	OR	β	*p* value	OR	β	*p* value
Nonperfusion grade	0.054	− 2.923	**0**.**008**	0.039	− 3.238	**0**.**006**
SSPiM in SVC	4.773	1.563	**0**.**033**			0.796
SSPiM in DVC	7.500	2.015	**0**.**018**	10.908	2.390	**0**.**013**

*OR* odds ratio, *SSPiM* suspended scattering particles in motion, *SVC* superficial vascular complex, *DVC* deep vascular complex.

### Sub-analysis of SSPiM after intravitreal injection

**Figure 1:**
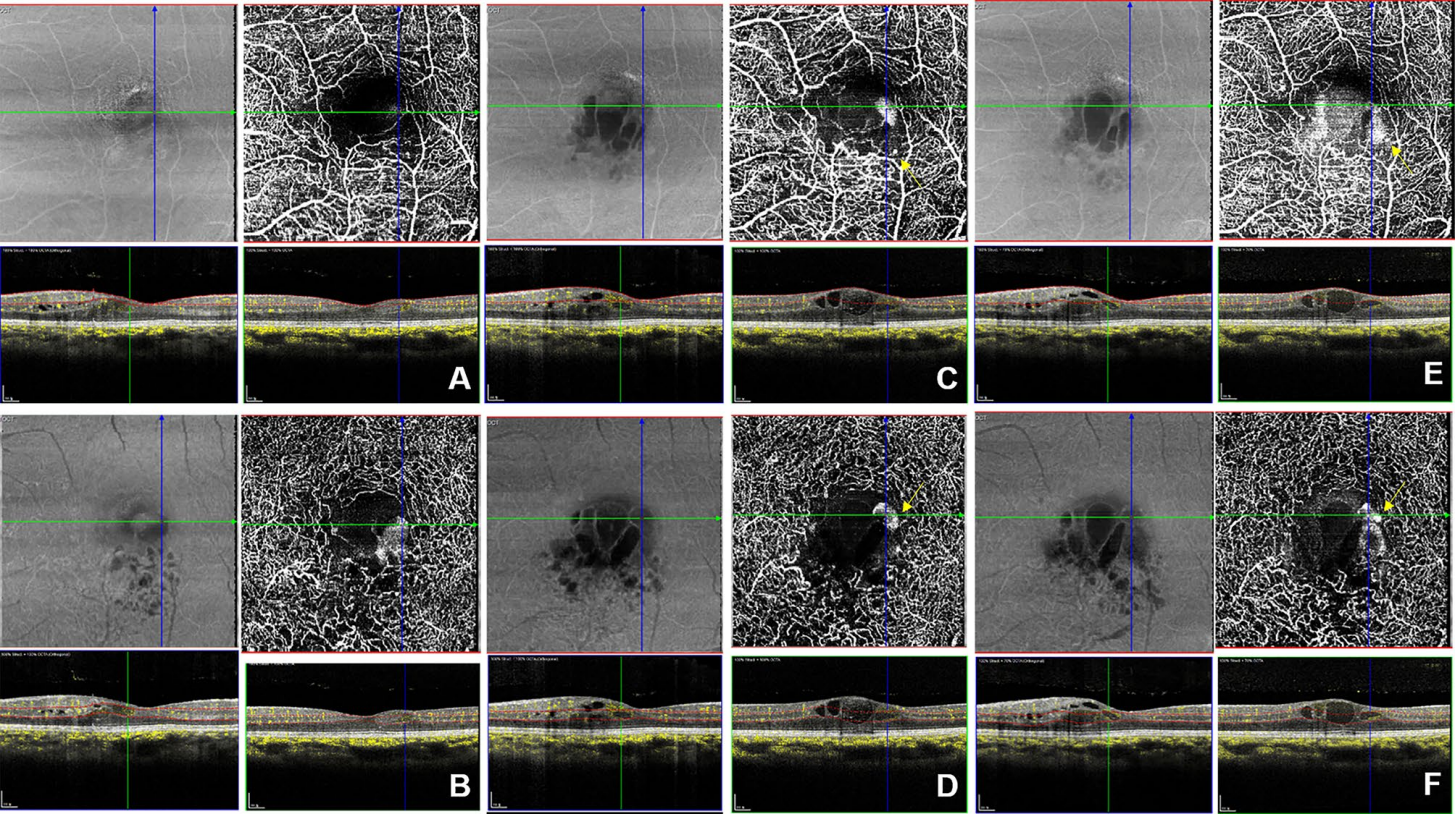
Sequential images of a case with a previous single injection and 5 month disease duration. During 12 months of follow-up, visual acuity remained better than 0.1 logMAR. (**A**,**B**,**C**,**D**,**E**,**F**) *En face* OCTA images (top right), *en face* OCT image (top left), vertical B-scan images with flow signal (bottom left), horizontal B-scan images with flow signal (bottom right). (**A**,**C**,**E**) Images of the SVC (top). (**B**,**D**,**F**) Images of the DVC (top). (**A**,**B**) SSPiM was first observed in both the SVC and DVC. (**C**,**D**) Three months later, images showed an increased area of SSPiM in both the SVC and DVC. Microaneurysms were observed near the SSPiM (yellow arrow, top right). (**E**,**F**) Six months later, images showed a further increase in area of the SSPiM in both the SVC and DVC as compared within earlier images (**B**), although there were no interval changes in cyst size between the images shown in (**E**) and (**F**) (bottom left). (**C**,**D**,**E**,**F**) Microaneurysms were also observed near the SSPiM (yellow arrow, top right). Photoshop CC 2017 (Adobe Systems Co, San Jose, CA) was used to create this figure.

**Figure 2:**
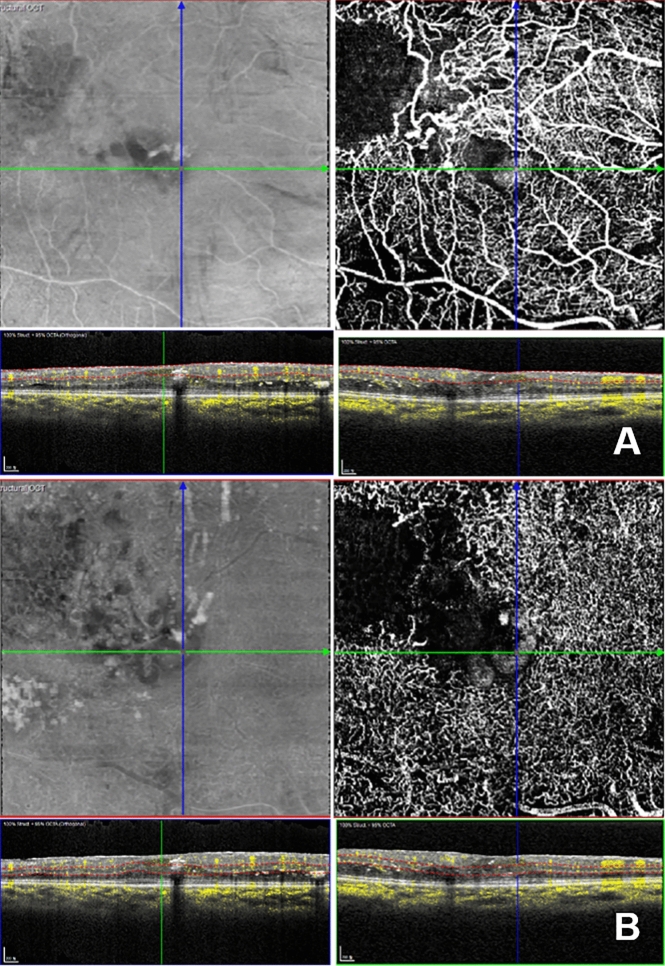
A representative case of SSPiM. This case shows a grade 1 nonperfusion area (axis is about 80 to 90 degree). (**A**,**B**) Top left is an *en face* OCT image. Top right is an *en face* OCTA image. The bottom left is the corresponding vertical B-scan image with flow signal. The bottom right is the corresponding horizontal B-scan image with flow signal. **A**. SSPiM with flow signal on the fovea was observed in the SVC (top right). (**B**) SSPiM with flow signal on the fovea was also observed in the DVC (top right). (**A**,**B**) Flow signals were observed in cystoid lesions corresponding to SSPiM (bottom). Photoshop CC 2017 (Adobe Systems Co, San Jose, CA) was used to create this figure.

Among 24 cases with SSPiM, only three cases resolved completely after one intravitreal Avastin injection while the other 21 cases received 1.58 ± 0.93 subsequent injections. Fourteen cases with persistent SSPiM after one intravitreal injection did not have larger CRT values than the 10 cases with transient SSPiM, but there were significant differences in the number of previous intravitreal injections (5.20 ± 1.32 vs 1.57 ± 1.39, *p* < 0.001), disease duration (26.00 ± 19.86 months vs 9.07 ± 11.88 months, *p* = 0.003), and disease-free duration (12.80 ± 11.02 months vs 5.50 ± 6.16 months, *p* = 0.036) between the sub-group with persistent SSPiM and the sub-group with transient SSPiM. During follow-up, cyst size decreased on OCT images in most patients, and this corresponded with SSPiM on OCTA images. Most SSPiM were located at the vascular–nonvascular junction (junction between the perfused retina and the nonperfused retina), even when multiple SSPiMs were simultaneously located in a single cyst (Fig. 1). As mentioned above, BCVA was not significantly different between groups 1 and 2. In one case, there was a consistent central cystoid lesion corresponding to SSPiM during 12 months of follow-up, but this patient’s visual acuity remained 20/20 (Fig. 2).

## Discussion

Nonvascular decorrelation signals can lead to challenges or difficulties in finding the real retinal microvasculature during OCTA image analysis. However, nonvascular decorrelation signals are not always artifacts and could have clinical and anatomical meaning^15–17,20^. In our study, we investigated if SSPiM in OCTA images had clinical relevance.

Cases with SSPiM (group 1) had a longer disease duration than cases without SSPiM (group 2). There were also significant differences in disease-free duration and previous injection number between the two groups. One hypothesis to explain SSPiM are that these are particles remaining after resolution of a retinal hemorrhage plus fluid leakage from the broken BRB following venous obstruction. Another hypothesis is that SSPiM are related to microaneurysms because microaneurysms in BRVO are related to chronic disease and are known to be factors for ME relapse^2,21,22^. Microaneurysms in BRVO can occur at the junction of the nonperfused and perfused retina^23^, and one study suggested that microaneurysms are a secondary change caused by local hypoxia and increased VEGF concentration^9^. Although these microaneurysms and SSPiM may be simultaneous occurrence and their postmortem relationship is unknown, we found that SSPiM were significantly associated with the presence of microaneurysms in the DVC in our multivariate analysis.

McLeod et al. showed that each capillary unit may serve as a Krogh cylinder^24^. Several studies in humans and animals have also demonstrated that retinal vasculatures, especially venules, may connect to the DVC without direct connection with the SVC and that the DVC may function as a venous route^6,7,25–27^. Furthermore, the outer plexiform layer comprises highly active metabolic tissue^28,29^. The middle retina may be more vulnerable to ischemia than the inner retina because the middle retina is far from both the choroidal and retinal circulations. In contrast, the inner retina has low oxygen demand because oxygen diffusion from the vitreous is also available. These results are consistent with the significant association between SSPiM of the DVC and treatment response and our finding that there were more microaneurysms in the DVC than in the SVC (although this difference was not statistically significant).

However, the results of the current study contradict some of our those of our previous study of SSPiM in DME. The factor most associated with treatment response in our previous study of DME patients was microaneurysms in the SVC. In DME, both the SVC and DVC have vascular diseases, and it appears that in these patients, the change in the SVC is larger than that in the DVC. Most SSPiM in BRVO cases in this study were observed in both the SVC and DVC. Abnormalities of the DVC may be more common than those of the SVC in vascular diseases such as BRVO. It is also possible that the SSPiM may sink toward the DVC while lying in bed at night because cysts corresponding to SSPiM on OCTA were composed of denser mass-like substances than cysts in DME. Furthermore, most BRVO cases with SSPiM showed more incomplete anatomical resolution than DME cases with SSPiM. Mean change in cyst size in the group with SSPiM before and after treatment was 43% in the current study. Further studies are needed to determine whether the positioning of the SSPiM changes as posture changes in both BRVO and DME patients.

Anatomical response of cysts to anti-VEGF injection was significantly related to nonperfusion grade and SSPiM in the DVC in multivariate analysis, while there were no significant factors related to the anatomical response of the CRT after anti-VEGF injection. Previous fundus autofluorescence studies showed that retinal nonperfusion was correlated with ME in BRVO cases^30,31^. In addition, foveal capillary nonperfusion on FAF images and macular thickness on OCT images were related to the recurrence of ME after intravitreal anti-VEGF treatments in BRVO^5^. Although our previous study focused on acute BRVO cases with ME, nonperfusion areas on OCTA images were consistently associated with ME recurrence^32^.

This study had several limitations. First, it was a retrospective, cross-sectional study with a small number of cases and therefore low statistical power. Due to the retrospective nature of the study, we were not able to assess the overall frequency of SSPiM. Furthermore, we did not find factors significantly related to CRT response. Additionally, we evaluated OCTA images using the Spectralis imaging system, while other OCTA analyses of BRVO cases used other OCA devices^8–10,12,16^. Differences in the settings for the SVC and DVC in different OCTA software programs may affect the *en face* OCTA images. Additionally, we only used one anti-VEGF drug (bevacizumab) for treatment. Further studies of other anti-VEGF drugs such as ranibizumab, aflibercept, triamcinolone, or dexamethasone are required to further evaluate the treatment response of BRVO patients with SSPiM. Last, we could not accurately evaluate vessel density and FAZ area because retinal hemorrhage and exudate were frequently observed in our BRVO patients.

In conclusion, we demonstrated that SSPiM on OCTA images are present in chronic BRVO cases and may be correlated with a poor response to intravitreal bevacizumab treatment. Nonperfusion grade in addition to SSPiM in the DVC were significantly associated with a poor anatomical response of cysts to treatment. Eyes with SSPiM had more microaneurysms in the DVC than eyes without SSPiM. Future prospective studies using one OCTA machine in acute BRVO patients with a long-term follow-up duration will be helpful for further elucidating the clinical relevance of SSPiM.

## Materials and methods

### Patients

The Institutional Review Board of Korea University Guro Hospital approved this study and waived the requirement to obtain signed consent for study participation. All research and data collection were conducted following the tenets of the Declaration of Helsinki.

As a retrospective cross-sectional study, chart review was done for consecutive patients diagnosed with unilateral BRVO with ME at Korea University Guro Hospital between March 2018 and May 2019. All patients received complete ophthalmic examinations at their visit, including best-corrected visual acuity (BCVA) measurement (expressed as logMAR units), slit-lamp examination, dilated fundus examination, and OCT and OCTA imaging. During follow-up, all examinations were performed again and analyzed. In addition, total disease duration, disease-free duration, blood pressure, and intraocular pressure (IOP) before intravitreal bevacizumab injection were reviewed. Disease-free duration was defined as the time interval between the previous injection period and the recurrence period. IOP was measured by Goldmann applanation tonometry. Systolic blood pressure (SBP) and diastolic blood pressure (DBP) were measured with a digital automatic blood pressure machine^33^. Mean ocular perfusion pressure (MOPP) was defined as 1/3 (mean arterial pressure—IOP) and mean arterial pressure (MAP) was defined as DBP + 1/3 (SBP−DBP)^33^.

Study inclusion criteria were as follows: (1) unilateral BRVO with cystoid ME; (2) treatment with one or more intravitreal bevacizumab injections; (3) OCTA images taken before and after intravitreal bevacizumab injection; (4) central retinal thickness (CRT) of ≥ 300 μm as measured by OCT before intervention; (5) OCT with a signal-to-noise ratio of 0.6 or greater; and (6) OCTA images of good quality (signal strength index > 30). The exclusion criteria were as follows: (1), Central retinal vein occlusion (CRVO); (2) follow-up period < 5 months after intravitreal anti-VEGF injection; (3) presence of other retinal diseases that could potentially affect the macula architecture; (4) pseudophakic eyes; (5) history of laser photocoagulation; and (6) OCTA images with artifacts such as motion artifacts of blinking artifacts.

### Optical coherence tomography and optical coherence tomography angiography

OCT and OCTA images were taken using a Spectralis OCT imaging device (version SP 6.7.21.0, central wavelength of 870 nm, speed of 85,000 A-scans/second, horizontal resolution of 5.7 μm, and axial resolution of 3.9 μm; Heidelberg Engineering, Heidelberg, Germany) at Guro Hospital.

OCTA examinations were done using a 3.0- by 3.0-mm volume scan pattern centered on the fovea. *En face* OCTA images were exported from the image viewer software (Heidelberg Eye Explorer, software version 1.21; Heidelberg Engineering, Heidelberg, Germany) after projection artifact removal^19^. *En face* OCTA images of the superficial vascular complex (SVC) and deep vascular complex (DVC) were generated based on automated layer segmentation.

### Image analysis

**Figure 3:**
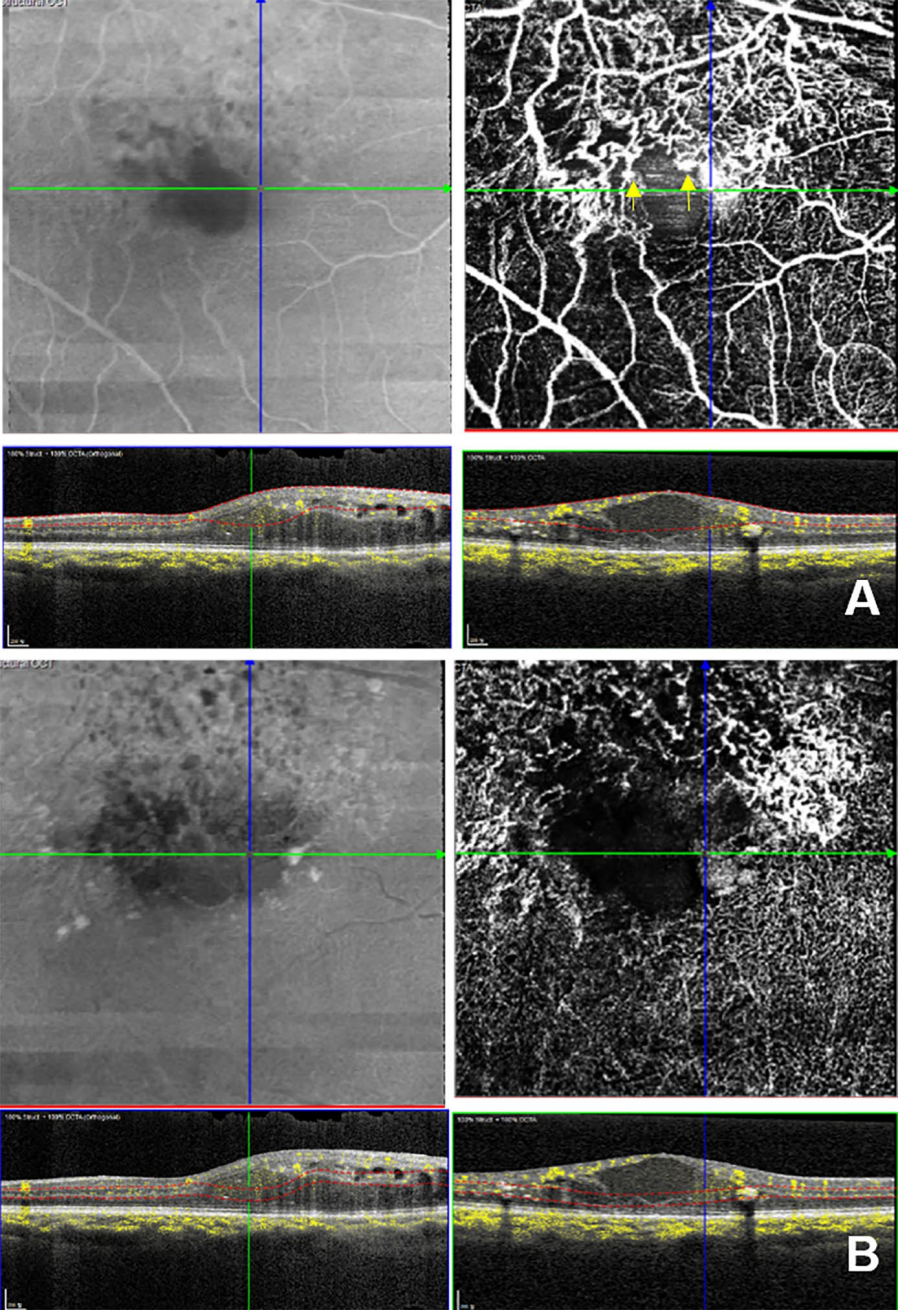
OCT and OCTA images of a case with five previous injections and 38-month disease duration. This case shows a grade 2 nonperfusion area (axis is about 150 to 160 degrees). (**A**,**B**) Top left is the *en face* OCT image. Top right is the *en face* OCTA image. The bottom left is the vertical B-scan with flow signal. The bottom right is the horizontal B-scan with flow signal. **A**. SSPiM with flow signal was observed at the vascular–nonvascular junction (junction between the perfused retina and the nonperfused retina) in the SVC (top right). Microaneurysms were observed near the SSPiM (yellow arrow, top right). (**B**) SSPiM with flow signal was observed at the vascular–nonvascular junction in the DVC (top right). Photoshop CC 2017 (Adobe Systems Co, San Jose, CA) was used to create this figure.

The Heidelberg image viewer software defined the SVC as the region from the internal limiting membrane to the outer border of the inner plexiform layer (IPL). The DVC was considered the area from the outer border of the IPL to the outer border of the outer plexiform layer. SSPiM in *en face* OCTA images was defined as a cystic lesion with a nonvascular decorrelation signal (Fig. 3). Cases with SSPiM were assigned to group 1, and cases without SSPiM were assigned to group 2. We could not evaluate the area of the foveal avascular zone and vessel density accurately due to cystic lesions on OCTA images; nevertheless, vessel density reduction can be used to predict whether recurrences of macular edema will develop after the initial anti-VEGF injection in BRVO cases^34,35^. OCTA images in our macular edema cases were not appropriate for accurate evaluate of vessel density and FAZ. Instead, we assessed the simplified nonperfusion grade. In our previous study of the nonperfusion area in BRVO using OCTA images, receiver operating characteristic curve analysis was used to identify the cutoff value; this was 3/8 of the area in the 3-mm zone, which had a sensitivity of 48% and specificity of 93.33%^32^. The degree of nonperfusion in the fovea 3-mm zone was divided into two grades according to the angle of the nonperfusion area (grade 1: < 135 axis degrees vs grade 2: ≥ 135 axis degrees) (Figs. 1 and 3). In addition, CRT and ME type were evaluated. CRT was defined as the mean distance between the upper border of the retinal pigment epithelium and the internal limiting membrane in a 1-mm diameter foveal zone. ME type was evaluated in the initial OCT images and classified as follows^5^. Serous retinal detachment was defined if subretinal fluid was present, while cystoid ME was defined as the presence of an intraretinal cystoid. The third classification type was mixed serous retinal detachment and cystoid ME. Microvascular abnormalities such as capillary telangiectasias, collateral vessels (venovenous drainage), and microaneurysms were also evaluated^9^. In particular, we investigated if microaneurysms were located near the cystoid ME or not.

Changes in intraretinal cyst size and CRT were also evaluated. Cyst responders were defined as cases where there was a reduction of ≥ 20% in preinjection cyst size after intravitreal injection. Cyst nonresponders were defined as those cases where there was cyst enlargement or a reduction in cyst size of < 20% after intravitreal injection. CRT responders were defined as patients with a reduction in CRT of ≥ 10% of preinjection CRT after intravitreal injection. CRT nonresponders were defined as those patients with enlargement of CRT or with a reduction in CRT of < 10% the preinjection CRT after intravitreal injection.

Images were graded independently by two retinal experts (K. E. C. and S. W. K.). Final grade was determined by consensus and was used in subsequent analyses.

### Statistical analysis

Baseline factors were compared between cases with SSPiM and those without SSPiM using the Mann–Whitney U test, chi-squared analysis (or Fisher’s exact test), Wilcoxon signed-rank test, or linear-by-linear test. Multivariate logistic analysis of significant parameters identified in the univariate analyses as independent variables was used to find significant factors associated with SSPiM. Statistical analyses were performed using SPSS version 21.0.0.0 (IBM, Armonk, NY, USA) or MedCalc V.12.1.3.0 (MedCalc Software bvba, Ostend, Belgium) software. All statistics were two-tailed and *p* values less than 0.05 were considered to be statistically significant.

## Data Availability

The datasets generated during and/or analyzed during the current study are available from the corresponding author on reasonable request.
